# Array of solid-state dye-sensitized solar cells with micropatterned TiO_2_ nanoparticles for a high-voltage power source

**DOI:** 10.1186/1556-276X-8-491

**Published:** 2013-11-20

**Authors:** Seong-Min Cho, Hea-Lim Park, Min-Hoi Kim, Se-Um Kim, Sin-Doo Lee

**Affiliations:** 1 School of Electrical Engineering, Seoul National University, Kwanak, PO Box 34, Seoul 151-600, South Korea; 2 School of Global Convergence Studies, Hanbat National University, 125 Dongseodaero, Yuseong-gu, Daejeon 305-719, South Korea

**Keywords:** Titanium dioxide nanoparticle, Micropatterning, Solid-state dye-sensitized solar cell, High-voltage source, 81.16.Rf, 85.40.Hp, 84.60.Jt

## Abstract

We demonstrate an array of solid-state dye-sensitized solar cells (SS-DSSCs) for a high-voltage power source based on micropatterned titanium dioxide nanoparticles (TNPs) as photoanodes connected in series. The underlying concept of patterning the TNP of a few micrometers thick lies on the combination of the lift-off process of transfer-printed patterns of a sacrificial layer and the soft-cure treatment of the TNP for fixation. This sacrificial layer approach allows for high pattern fidelity and stability, and it enables to construct stable, micrometer-thick, and contamination-free TNP patterns for developing the SS-DSSC array for miniature high-voltage applications. The array of 20 SS-DSSCs integrated in series is found to show a voltage output of around 7 V.

## Background

Dye-sensitized solar cells (DSSCs) with mesoporous titanium dioxide (TiO_2_) nanoparticles (TNPs) have been considered as a promising alternative to conventional inorganic solar cells due to their relatively high power conversion efficiencies and low production cost [1]. So far, much effort has been made toward the enhancement of the power conversion efficiency of the DSSCs [2-4]. Together with the improvement of the power conversion efficiency, the generation of high output voltage is one of the critical issues for practical applications. The issue of the high voltage generation of the DSSCs has been addressed only in a unit cell producing limited output voltages of around 1 V [5-7], which is far below the voltages required for most practical devices, for example, around 4 V for mobile phones. Thus, the integration of DSSCs needs to be pursued for high-voltage sources. Owing to the excellent electron transport characteristics, stability, and appropriate conduction band position, a TNP layer is promising for use as a photoanode in the DSSC [8]. Therefore, for the integration of a DSSC array, a reliable patterning technique of the TNP layer should be developed.

In patterning the TNP, several methods such as solvent-assisted soft lithography [9], micromolding technique in capillaries [10], and imprint lithography [11] have been typically employed, but they involve the difficulty of patterning multiple stacks of the TNP and eliminating the residual layer. In other words, these patterning methods are not applicable for constructing relatively thick (a few micrometers) and stable TNP patterns demanded for sufficiently high absorption of light in the DSSCs [12]. Moreover, the DSSCs with liquid electrolytes encounter confinement problem, leakage, and evaporation of the liquid in the integration into the array. Therefore, it is extremely important to develop a versatile method of patterning a few-micrometer-thick TNP layer for fabricating an array of solid-state dye-sensitized solar cells (SS-DSSCs).

In this work, we demonstrate an array of SS-DSSCs for a high-voltage power source using micropatterned TNP as photoanodes connected in series. The basic concept relies primarily on a chemically compatible lift-off process of a fluorous sacrificial layer which has the complementary patterns of the TNP of a few micrometers thick on a substrate. This sacrificial layer approach allows for high pattern fidelity and stability, and it leads directly to stable, micrometer-thick, and contamination-free TNP patterns for developing the SS-DSSC array for miniature high-voltage applications.

## Methods

### Fabrication of TNP patterns

In preparing photoanodes connected in series for a high-voltage DSSC array, micropatterns of the TNP were constructed on a pre-patterned fluorine-doped tin oxide (FTO) glass. An array of 20 FTO electrodes, where each electrode has a width of 500 μm and a gap of 500 μm between two adjacent electrodes, was prepared using photolithography and a dry etching process. A glass substrate with pre-patterned FTO was cleaned with acetone, deionized water, and ethanol in sequence and dried with nitrogen flow. The cleaned substrate was then dried at 90°C in a vacuum oven for 10 min to remove any residual water and subsequently treated with ultraviolet ozone for 5 min. In order to improve the adhesion and the mechanical strength of the TNP layer [13], the treated FTO glass was soaked in an aqueous solution of 40 mM TiCl_4_ at 70°C for 30 min. The FTO glass was then cleaned in the same way described above.

**Figure 1:**
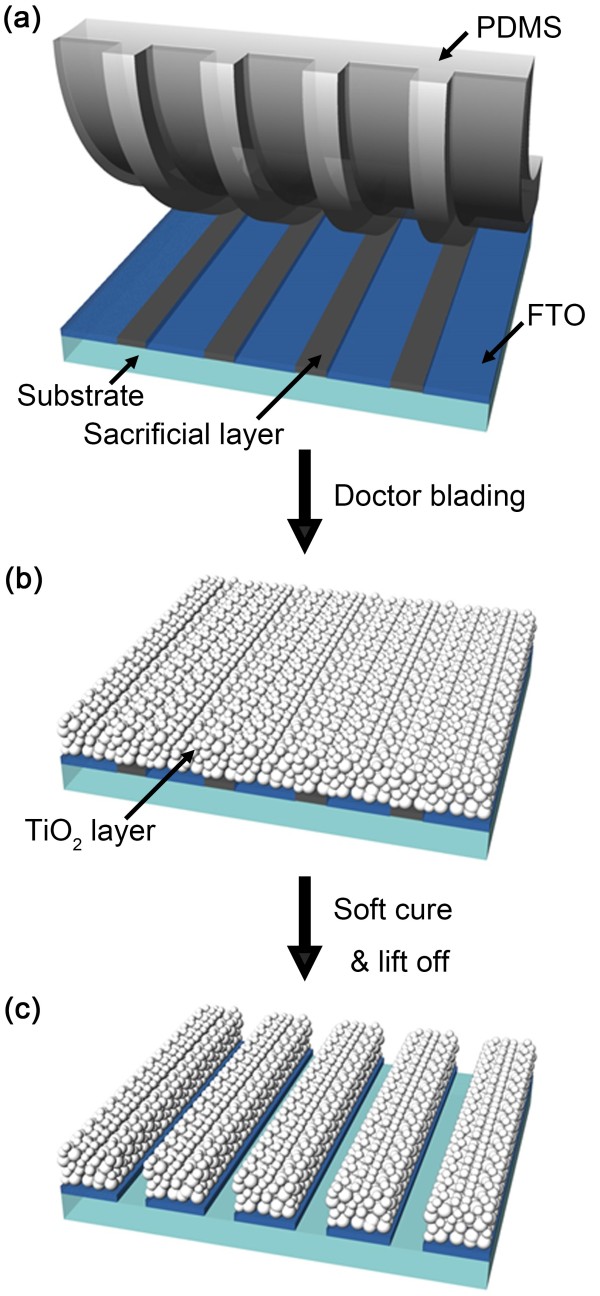
**Schematic diagram showing each step of our micropatterning method of TNPs. (a)** Transfer printing of the SL on a patterned FTO glass using a PDMS stamp. **(b)** Doctor-blading TNP paste on the SL-patterned FTO glass to form a TNP layer of 2.5 μm thick. **(c)** Soft-curing of the TNP layer at 50°C for 3 min and the lift-off process of the SL.

**Figure 2:**
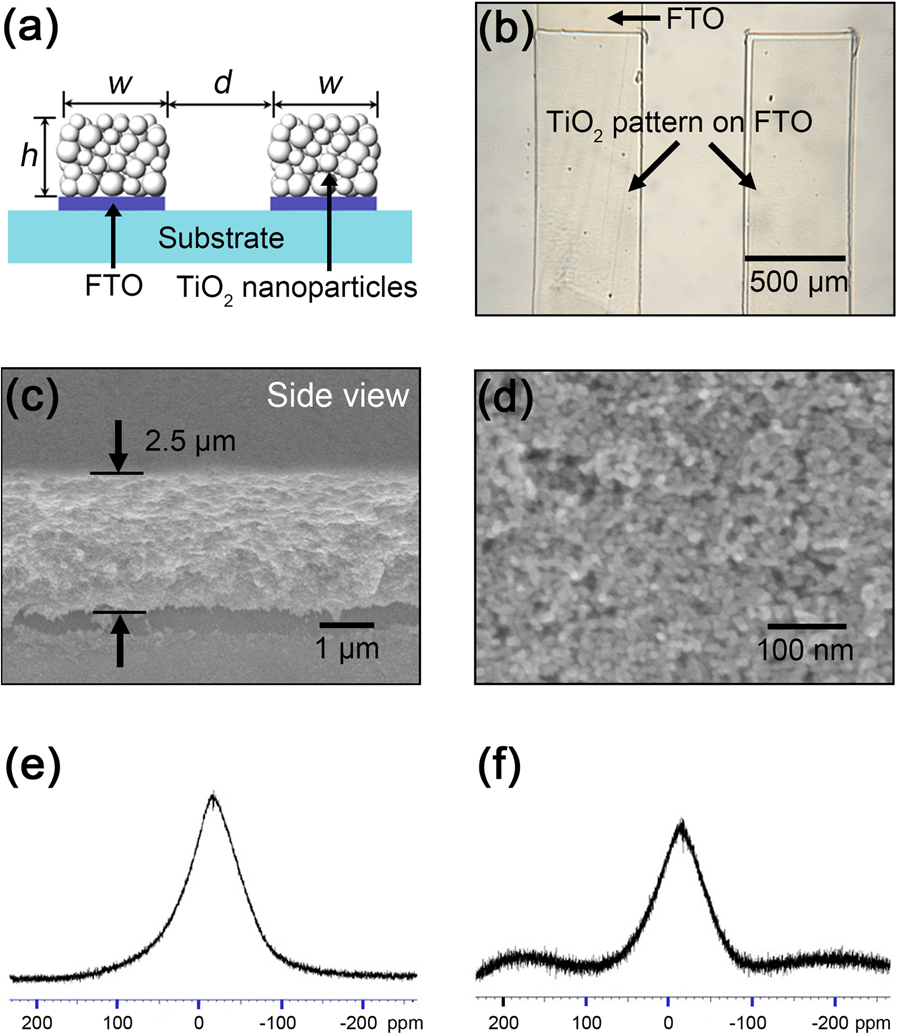
**Schematic diagram of TiO**_**2 **_**pattern, images taken with optical microscopy and FE-SEM, and solid **^**19**^ **F-NMR spectra. (a)** Dimension of a TiO_2_ pattern: the width (*w*), the distance (*d*), and the height (*h*) are 500, 500, and 2.5 μm, respectively. **(b)** The optical microscopic image of the TNP patterns on the FTO glass. **(c)** The FE-SEM image of the cross section of the patterned TNP layer of 2.5 μm thick. **(d)** The high-resolution FE-SEM image of the highly packed TNPs. The solid ^19^ F-NMR spectra of **(e)** an empty rotor and **(f)** a TNP layer after being treated with a fluorous solvent.

Figure 1 shows the schematic diagram illustrating the fabrication of a patterned TNP layer on the FTO glass. The entire fabrication processes of patterning TNP are as follows: An elastomer stamp with patterns, complementary to desired TNP patterns, was made of poly-(dimethylsiloxane) (PDMS). For fabricating complementary patterns of a sacrificial layer (SL) on the FTO glass, a fluorous polymer (3 M Novec™ EGC-1700, 3 M Novec, Manassas, VA, USA) dissolved in a highly fluorous solvent (3 M Novec™ HFE-7100) was dip-coated on the prepared PDMS stamp. Figure 1a shows the transfer printing process of the complementary patterns of the SL on the PDMS stamp onto the FTO glass. Note that no additional pressure or heat is required during transfer printing due to the lower surface energy of the PDMS stamp than that of the FTO glass [14]. Ti-Nanoxide T (Solaronix SA, Aubonne, VD, Switzerland) paste was subsequently prepared on the SL-patterned FTO glass to form a TNP layer using a doctor-blading technique, as shown in Figure 1b. The TNP film was soft-cured at 50°C for 3 min for the fixation of the TNPs to ensure stability during the following lift-off process. In the soft-cure treatment, the duration of heating plays a critical role in patterning the TNP layer of a few micrometers thick; the TNP layer should be sufficiently soft for the application of the lift-off process but structurally strong enough to prevent the collapse of the TNP stacks during the lift-off process. Figure 1c illustrates the SL layer which was lifted-off in a fluorous solvent, leaving only the line patterns of the TNP layer on the FTO active regions. Note that the fluorous solvent is chemically inert to most organic and inorganic materials [14,15]. The patterned TNP layer was annealed at 80°C for 2 h and then at 450°C for 30 min. As shown in Figure 2a, the TNP pattern whose width (*w*) and distance (*d*) were 500 μm, respectively, was well defined according to the PDMS pattern. In principle, the TNP patterns can be achieved down to a submicrometer scale depending on the dimension of the elastomer stamp patterns or the SL patterns [11].

### Preparation of a DSSC array

Each patterned TNP used as an individual photoanode for a unit cell was connected in series for a high-voltage DSSC array. The patterned TNP layer was immersed in a solution of 3 mM Z907 dye (Solaronix SA) dissolved in a 1:1 mixture of acetonitrile and *tert*-butyl alcohol for 24 h. The dye-coated TNP layer was simply washed with acetonitrile. For the solid-state hole transport material (HTM), spiro-OMeTAD (American Dye Source, Inc., Baie D'Urfé, Quebec, Canada) dissolved in chlorobenzene was mixed with a lithium bis(trifluoromethylsulfonyl)imide salt ionic dopant dissolved in acetonitrile. The solution was placed on the whole TNP-patterned FTO glass, and the pores in the TNP layer were filled with the solution by capillary action for 1 min. The TNP-patterned FTO glass was then spun at the rate of 2,000 rpm. For the preparation of a cathode, Au of 100 nm thick was thermally deposited at the rate of 1 Å/s through a shadow mask to connect 20 cells in series. The array of 20 DSSCs connected in series has a total active area of 1.4 cm^2^.

### Characterization methods

An optical microscope and a field emission scanning electron microscope (FE-SEM; SU-70, Hitachi, Ltd., Chiyoda, Tokyo, Japan) were used for taking the images of the patterned TNP layer. In order to examine the existence of any residual fluorous solvent in the patterned TNP layer which may deteriorate the photovoltaic performance of the SS-DSSCs, solid fluorine-nuclear magnetic resonance (^19^ F-NMR) spectra were measured with a Bruker AVANCE II (500 MHz) spectrometer (Bruker, Billerica, MA, USA) with a 2.5-mm probe at the spin rate of 20 kHz. A current–voltage curve was obtained using a source measure unit (model 2400, Keithley Instruments Inc., Cleveland, OH, USA) under the illumination of a solar simulator with air mass 1.5 global (AM 1.5 G) filters at 100 mW/cm^2^. The light intensity of the solar simulator was calibrated with a standard silicon diode.

## Results and discussion

The optical microscopic image of the TNP patterns in the FTO regions on the substrate is shown in Figure 2b where TNP patterns isolated from the neighboring patterns were clearly seen. Each isolated TNP pattern, which is 500 μm wide and 14 mm long in the interval of 500 μm, represents an individual photoanode for a unit cell in the SS-DSSC array [14,15]. Figure 2c shows the FE-SEM image of the cross-sectional TNP pattern. According to the FE-SEM image, each TNP pattern was about 2.5 μm thick. This is a typical thickness of the TNP photoanode for a whole SS-DSSC [12]. Moreover, as shown in Figure 2d, the TNPs were highly packed in the multistacks of a few micrometers, and the surface roughness was about a few tens of nanometers. It should be noted that our micropatterning method based on the SL lift-off process is very simple and effective to produce a wide range of the TNP patterns by varying the thickness of the doctor-bladed TNP layer and the dimension of the SL patterns transfer-printed by the PDMS stamp.

For lifting-off the SL, the FTO substrate with the TNP patterns was exposed to a fluorous solvent. From the measurements of the ^19^ F-NMR spectrum of the TNP sample treated by a fluorous solvent, no extra peak was observed when compared to an empty rotor, as shown in Figure 2f. This tells us that no remnant solvent exists after annealing the TNP sample at 450°C, and thus, the SL lift-off process is contamination free for patterning the multistacks of TNPs in the fabrication of the array of the SS-DSSCs.

**Figure 3:**
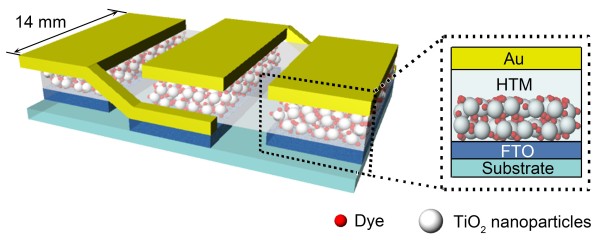
Schematic diagram showing an array of three SS-DSSCs connected in series and a unit cell.

Figure 3 shows the array configuration of three DSSCs connected in series together with a cross-sectional view of a unit cell consisting of the FTO layer, TNPs with dyes, HTM, and Au electrode. For the series connection, the Au cathode in a certain unit cell is connected to the patterned FTO layer in the adjacent unit cell. In describing the charge flow in the unit DSSC, when the incoming light is absorbed by the photosensitizing dyes, the electrons are injected into the conduction band of the TNPs and move toward the FTO electrode. Meanwhile, the oxidized dyes are reduced by the HTM which is regenerated at the Au cathode [16].

**Figure 4:**
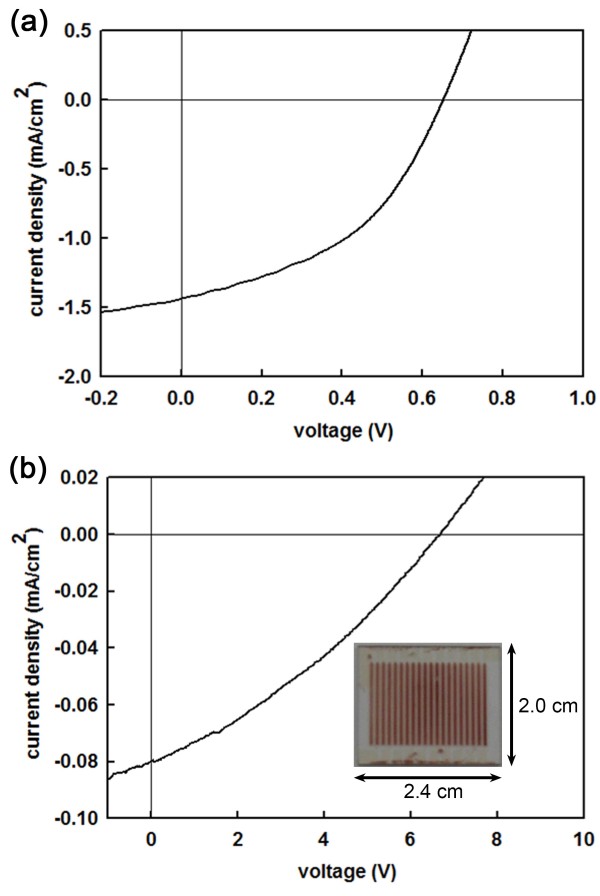
**Current–voltage curves of SS-DSSCs.** Current–voltage curves of **(a)** a single cell and **(b)** an array of 20 SS-DSSCs measured under the illumination of a simulated AM 1.5 G solar light (100 mW/cm^2^). The inset shows the fabricated array of 20 SS-DSSCs with a total length of 2.0 cm and width of 2.4 cm.

Figure 4a,b shows the current–voltage curve of a single SS-DSSC and that of the array consisting of 20 SS-DSSCs measured under the illumination of simulated AM 1.5 G solar light (100 mW/cm^2^). For a single cell, the values of the short-circuit current density (*J*_sc_), open-circuit voltage (*V*_oc_), and fill factor (FF) are found to be 1.44 mA/cm^2^, 0.65 V, and 0.44, respectively. The power conversion efficiency (PCE) is about 0.41%. For the array of 20 cells, the values of *J*_sc_, *V*_oc_, and FF are 0.08 mA/cm^2^, 6.68 V, and 0.32, respectively, and the resultant PCE is 0.17%. The series resistance (*R*_s_) of the single cell and that of the array of 20 cells derived from the inverse slopes of the plots (or d*V*/d*J* when *J* = 0) [17] are 1.52 × 10^2^ and 5.45 × 10^4^ Ω cm^2^, respectively. Note that the value of *V*_oc_ (6.68 V) for the array of 20 cells is quite smaller than the value (13 V) corresponding to the simple addition of *V*_oc_ for a single cell. This is partially attributed to the non-ideal series connection due to the non-patterned HTM. In addition, the alignment between FTO and the patterned TNP layer may not be perfect, and thus, the active regions become reduced. A better alignment would give a higher voltage. The values of the FF and the PCE also become low, due to the increase in the leakage current around the sides of the unit cells and the large value of *R*_s_ associated with more FTO-TNP interfaces and HTM-metal junctions. The photovoltaic performance can be improved, in principle, by tailoring the materials themselves, patterning the solid-state electrolyte, aligning accurately the FTO and the TNP patterns, and optimizing device parameters and geometries. It should be emphasized that our work provides a new route to the construction of TNP patterns of a few micrometers thick in a simple and reliable way.

## Conclusions

We presented how a functional layer of the nanoparticles can be patterned for use in hybrid electronic and optoelectronic devices in a simple, cost-effective, and contamination-free way. The underlying concept comes from the lift-off process of the transfer-printed patterns of a fluorous sacrificial layer and the soft-cure treatment of the nanoparticles for fixation. As an example, an array of the SS-DSSCs with a micropatterned TNP layer of several micrometers thick was demonstrated for high-voltage source applications. The array of 20 SS-DSSCs connected in series showed an open-circuit voltage exceeding 6 V. It is concluded that the micropatterning approach presented here will be applicable for a wide range of diverse nanoparticles to be employed in optical, electronic, and sensing devices.

## Abbreviations

AM 1.5 G: Air mass 1.5 global; DSSC: Dye-sensitized solar cell; FE-SEM: Field emission scanning electron microscope; FF: Fill factor; ^19^F-NMR: Fluorine-nuclear magnetic resonance; FTO: Fluorine-doped tin oxide; HTM: Hole transport material; *J*_sc_: Short-circuit current density; PCE: Power conversion efficiency; PDMS: Poly-(dimethylsiloxane); *R*_s_: Series resistance; SL: Sacrificial layer; SS-DSSC: Solid-state dye-sensitized solar cell; TiO_2_: Titanium dioxide; TNP: Titanium dioxide nanoparticles; *V*_oc_: Open-circuit voltage.

## Competing interests

The authors declare that they have no competing interests.

## Authors’ contributions

SMC, MHK, and SDL conceived and designed the experiment. SMC and SUK fabricated the TNP patterns. SMC and HLP fabricated the DSSC array, performed the electrical and optical measurements, analyzed the data, and interpreted the results. HLP, MHK, and SDL wrote the paper. All authors read and approved the final manuscript.
